# Evolutionary Characterization of the Pandemic H1N1/2009 Influenza Virus in Humans Based on Non-Structural Genes

**DOI:** 10.1371/journal.pone.0056201

**Published:** 2013-02-13

**Authors:** Chengmin Wang, Yanyu Zhang, Bin Wu, Shelan Liu, Ping Xu, Yanmin Lu, Jing Luo, Dale Louis Nolte, Thomas Jude Deliberto, Mingxing Duan, Hong Zhang, Hongxuan He

**Affiliations:** 1 National Research Center For Wildlife Born Diseases, Key Laboratory of Animal Ecology and Conservation Biology, Institute of Zoology, Chinese Academy of Sciences, Beijing, China; 2 National Wildlife Research Center, USDA/APHIS/Wildlife Services, United States Department of Agriculture, Fort Collins, Colorado, United States of America; 3 State Key Laboratory of Biomembrane & Membrane Biotechnology, School of Life Sciences, Tsinghua University, Beijing, China; 4 Department of Infectious Diseases, Zhejiang Center for Disease Control and Prevention, Binjiang District, Hangzhou, Zhejiang Province, China; 5 Beijing Institute of Transfusion Medicine, Academy of Military Medicine Sciences, Beijing, P.R China; Columbia University, United States of America

## Abstract

The 2009 influenza pandemic had a tremendous social and economic impact. To study the genetic diversity and evolution of the 2009 H1N1 virus, a mutation network for the non-structural (NS) gene of the virus was constructed. Strains of the 2009 H1N1 pandemic influenza A virus could be divided into two categories based on the V123I mutation in the NS1 gene: G1 (characterized as 123 Val) and G2 (characterized as 123 Ile). Sequence homology analysis indicated that one type of NS sequence, primarily isolated from Mexico, was likely the original type in this pandemic. The two genotypes of the virus presented distinctive clustering features in their geographic distributions. These results provide additional insight into the genetics and evolution of human pandemic influenza H1N1.

## Introduction

Within several months of its emergence in March 2009 in Mexico, a novel H1N1 influenza A virus of swine origin had spread throughout the world and caused the first influenza pandemic of the 21^st^ century. Like most seasonal influenza viruses, this new virus has been associated with only a mild self-limiting illness in the majority of people, although some populations (e.g., the young and those with certain chronic health conditions) are particularly susceptible to severe complications [1].

The genome of the influenza A virus contains 8 separate RNA segments coding for different proteins that play specific roles in the replication of the virus. Among these, non-structural proteins NS1 and NS2 are coded by the eighth segment of the viral genome, which contains 890 nucleotides (nt) [2]. The NS1 and NS2 genes have two overlapping sequences consisting of a 56-nt leader sequence containing the initiation codon (AUG) before the intron and a 210-nt sequence after the intron [2]. The NS2 protein is involved in viral assembly, providing a nuclear export signal and a binding region for the M1 protein [3]. The crystal structure of the C-terminal (M1-binding) domain of NS2 exhibits a helical hairpin that is amphipathic in nature, with one face being hydrophobic and the other hydrophilic [4].

A global effort is underway to control H1N1 in humans and to prevent human exposure, both of which may also reduce the risk of pandemic emergence. To better assist in the development and implementation of public health control measures involving diagnosis, immunization and antiviral drug therapy, we assessed the genetic diversity and characterized the evolution of the pandemic H1N1/2009 influenza A virus in humans.

## Materials and Methods

### 2.1 Sequence data

In addition to the sequence data we analyzed for the 2009 novel H1N1 virus, data were also obtained from the influenza sequence database (Influenza Virus Resource, http://www.ncbi.nlm.nih.gov/genomes/FLU/FLU.html, accessed May 14, 2010) [5]. Sequence data were included for all human and swine influenza A viruses with a full-length H1N1 subtype NS gene. A total of 728 pandemic 2009 H1N1 viruses were analyzed (Table S1, S2). A multiple alignment of nucleotide sequences was constructed using Clustal W.

### 2.2 Phylogenetic analyses

NS gene sequences from 728 human H1N1 viral strains and some reference strains were obtained from the NCBI Resources website Phylogenetic analyses were performed using the neighbor-joining method with 1,000 bootstraps and the maximum-likelihood method with 100 bootstraps in PHYLIP version 3.67 (http://evolution.gs.washington.edu/phylip.html) [6], and a network of mutations with different time nodes was constructed for the NS sequences using NETWORK version 4.5.1.0 (http://www.fluxus-technology.com/) [7]. Based on the tree structure, the swine viruses of the North American lineage, including triple reassortant viruses from which the 2009 novel H1N1 virus originated [8], [9], were selected for further analyses.

### 2.3 Selection pressure

Phylogenetic trees were constructed for the datasets of each host using the maximum-likelihood method implemented in PhyML-aLRT [10] with the General Time-Reversible (GTR) model. The GTR model included four rate categories, all parameters of which were estimated from the data. Positive selection sites were detected using the fixed effects likelihood method, which is based on maximum likelihood estimates. Relative rates of non-synonymous and synonymous substitutions (dN/dS) in each codon were compared. The sites where dN/dS>1 and dN/dS<1 were inferred as being positively and negatively selected, respectively. Furthermore, the overall strength of selection was calculated by comparing the global estimates, ω, of dN and dS averaged over the entire alignment [11]. Details of the method are described elsewhere [11], [12], [13]. The evolutionary pressure differential was also analyzed using HyPhy, which tests the hypothesis that the dN/dS ratio at a given site differs between two datasets along a phylogenetic tree. The details are described elsewhere [11], [12].

## Results

### 3.1 The NS2 gene showed positive selection pressure in April and August of 2009

**Figure 1:**
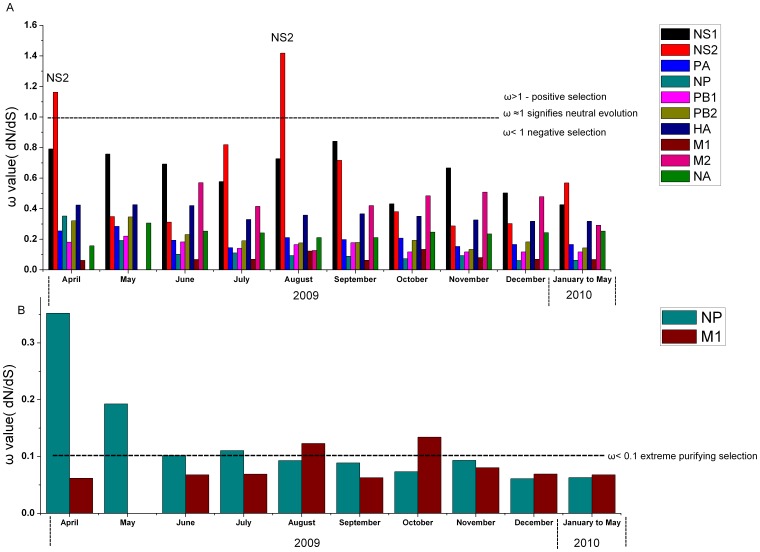
Selection pressure. Selection pressures on the whole sequence (ω) are calculated for the entire coding regions of the NS1, NS2, PA, NP, PB1, PB2, NA, HA, M1, and M2 genes of the novel swine-origin influenza virus A (H1N1) from April 2009 to May 2010. The number of gene sequences used in this study is shown in Table S1. The ratio ω = *dN/dS* has become a standard measure of selective pressure; ω≈1 signifies neutral evolution, ω<1 indicates negative selection, ω>1 indicates positive selection, and ω≤0.1 indicates extreme purifying selection. These results indicate that the NS2 gene was under positive selection in April and August 2009 but was under negative selection from May to July 2009 and from September 2009 to May 2010. The remaining eight genes were under negative selection from April 2009 to May 2010.

The ratio (ω) of nucleotide substitutions may indicate whether selection is occurring, as well as the degree and type of selection. The ratio (ω) has been used as a measure of evolutionary change and has become a standard measure of selective pressure [14]. Under neutral evolution, ω≈1, *dN*≈*dS*; any deviation of *dN* from *dS* may be due to positive Darwinian selection when ω>1.0 or may be due to purifying (stabilizing) selection when ω<1.0. Most genes exhibit a pattern of purifying selection (0.1<ω<1.0); however, the intensity of purifying selection varies for different genes. Very small changes in ω have biological relevance, and the greatest intensity of purifying selection occurs as ω approaches zero [15]. Genes where amino acid residues are critical for protein structure and function are expected to be coded for by codons that are under “extreme purifying selection”. We define extreme purifying selection as ω≤0.1. We calculated the selection pressures on the entire coding regions of ten viral genes (PB2, PB1, PA, HA, NP, NA, M1, M2, NS1 and NS2) of the human pandemic 2009/H1N1 influenza virus (Table S3). Here, we found that the NS2 genes were under positive selection in April (ω = 1.16193) and August (ω = 1.41777) of 2009, but were under negative selection from May to July 2009 and September 2009 to April 2010. The greatest number of genes were under negative selection from April 2009 to April 2010 (Figure 1A), but NP (ω≤0.1 from August 2009 to May 2010) and M1 (ω≤0.1, April to July 2009, September 2009 and from November 2009 to May 2010) were under extreme purifying selection (Figure 1B).

### 3.2 Two prevalent genotypes of 2009 influenza A (H1N1) based on the NS gene

**Figure 2:**
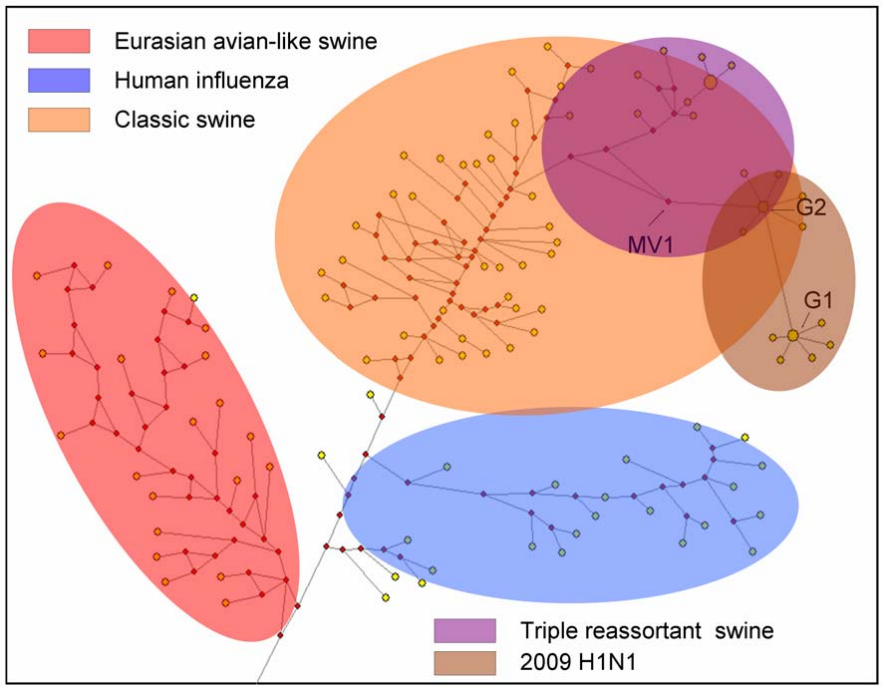
Mutation network for the NS gene of the H1N1 influenza viruses. A mutation network for Eurasian-“avian-like” swine (Red), classic swine (Orange), human influenza (Blue), triple reassortant swine (Violet), and 2009 H1N1 (Brown). The area of each node is in proportion to the number of sequences the node represents. The ancestral node representing the original sequence type (MV1) is indicated by a black arrow.

A network of mutations for the NS sequences was constructed using the NETWORK program with different time nodes (Figure 2). Every node in the network represents a sequence type observed in the 728 full-length sequences. A BLAST search for the nucleotide sequences in the NCBI database and homology analysis with an out-group (viruses from humans) indicated that a node containing many sequences could be treated as an ancestral node for all the observed sequences. The mutation network analysis indicated that MV1 might be an ancestral strain of the 2009 H1N1 influenza virus (Figure 2).

### 3.3 Different geographical distributions of two genotypes

**Figure 3:**
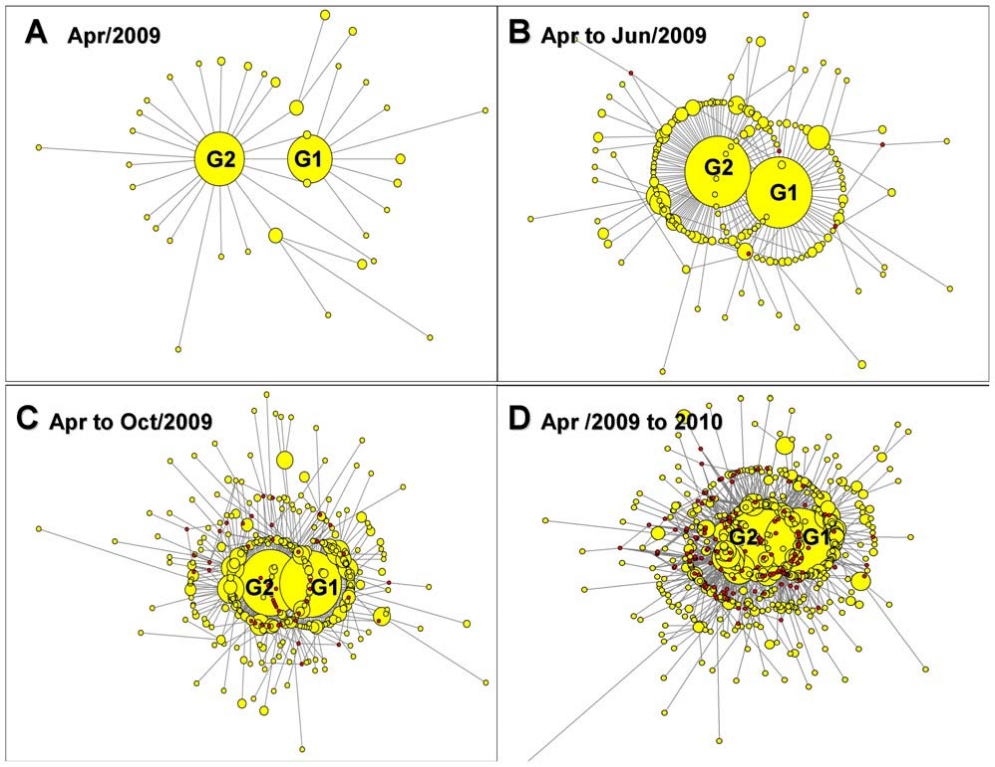
Mutation network for the NS gene of the novel influenza virus A (H1N1). Mutation networks for the NS gene on Apr 2009 (A), Apr to Jun 2009 (B), Apr to Oct 2009 (C), and Apr 2009 to May 2010 (D). The trend of the two prevalent genotypes (G2 and G1 type) remained unchanged from April 2009 to May 2010.

**Figure 4:**
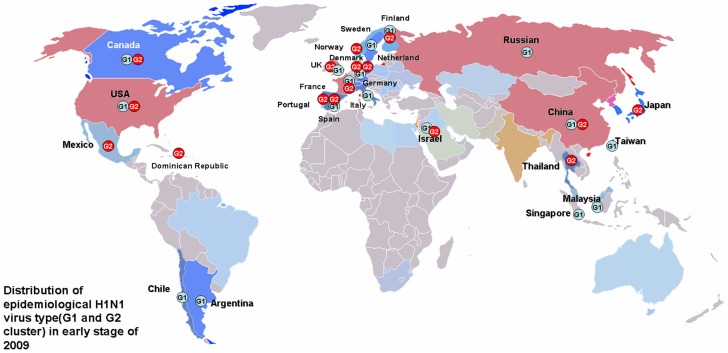
Worldwide distribution of G1 (A) and G2 (B) type H1N1 viruses. The G1 genotype was found in early April 2009 throughout North America, Israel, and Portugal. Patients infected in late April and May 2009 with the Mexico type were observed in other countries (e.g., Norway, Thailand, Denmark, the Dominican Republic, Spain, Taiwan, the Netherlands, the United Kingdom, Japan, China, and France). The G2 genotype was also found in the USA, Canada, and Israel, as well as in Taiwan, Malaysia, Singapore, Chile, Russia, Finland, China, Italy, France, and Sweden).

Based on the 123V and 123I mutations in the NS1 gene, the viruses evolving into the pandemic strain could be divided into two categories: the Mexico type (G2 type) and the New York type (G1 type), respectively. The trend of the two prevalent genotypes (G2 and G1 types) remained unchanged from April 2009 to May 2010 (Figure 3A to D). The G1 and G2 genotypes of the pandemic H1N1/2009 human influenza A virus originated from different countries or regions. This viral genotype, which was observed from late April 2009 and May 2010, had been detected in other countries (e.g., Norway, Thailand, Denmark, the Dominican Republic, Spain, Taiwan, the Netherlands, the United Kingdom, Japan, China, and France) (Figure 4 and Figure S1). The G1 type was found in the USA, Canada, and Israel, as well as in Taiwan, Malaysia, Singapore, Chile, Russia, Finland, China, Italy, France, Sweden, Germany, Spain, and Argentina (Figure 4 and Figure S1).

## Discussion

In this study, we extracted all gene sequences of the human pandemic H1N1/2009 influenza A virus strains (from April 2009 to May 2010) and some H1N1 reference strains from the NCBI website. High selection pressure indicated that a gene or site was under strong selection, i.e., positive selection for the amino acid substitution. Lower selection pressure indicated that a gene or site was under negative selection, i.e., retained the same amino acid(s) because changes might lead to proteins with reduced or no functionality [11], [16]. A dN/dS ratio >1 is evidence of positive natural selection [17], [18]. Here, the NS2 genes were under positive selective pressure in April (ω = 1.16193>1) and August 2009 (ω = 1.41777>1) but were under negative selective pressure from May to July 2009 and from September 2009 to April 2010. One possible explanation for this result is that the NS gene of the novel H1N1 virus had not yet fully adapted to humans. It has been suggested that the virus will continue to evolve towards greater viral fitness through mutations [19]. There has been considerable interest in possible selective factors acting on this gene because the NS protein is an effective interferon antagonist. Although NS genes and proteins are important in the life cycle of influenza viruses, it is not yet clear whether high selective pressure on NS2 in the early (April 2009) and middle (August 2009) stages of the pandemic was related to the 2009 outbreaks and its subsequent worldwide spread in humans.

The average value of ω for NP and M1 in 2009 and 2010 was much lower (ω≤0.1) than the average value of ω for the other genes; however, all the other genes, except for NS, were under negative selection from April 2009 to May 2010. These results suggest that the NP and M1 genes experienced significantly stronger purifying selection in 2009 and 2010 compared to other genes. However, NS2 experienced significantly stronger positive selection during the early and middle stages of the 2009 pandemic and may be important for understanding the etiology of the present pandemic influenza virus. These proteins are involved in regulating the balance between transcription and replication during the virus cycle [20], [21], [22], [23] and play a significant role in viral pathogenesis [24].

A maximum likelihood phylogenetic tree based on the nucleotide sequences of the NS gene of selected influenza viruses exhibited clusters corresponding to three major lineages: classical swine H1N1 (CS), European “avian-like” H1N1 (EA), and human season H1N1 viruses (data not shown). The 2009 H1N1 virus originated from triple-reassortant H1N1 (TRIG) [8]. Furthermore, a network of mutations for the NS sequences in our current study showed that the 2009 H1N1 virus belonged to the triple-reassortant H1N1 strain of the classical swine H1N1 lineage and could be divided into two different genotypes (G1 and G2) based on the 123V and 123I mutations in the NS1 protein. The mutation network provided evidence that the ancestral sequence type (MV1) originated from triple-reassortant H1N1 (TRIG) and appeared prior to the spread of the virus to a human host. Meanwhile, the 2009 virus evolving into the pandemic strain could always be divided into two major derivative categories: the G2 and G1 types.

It is unclear whether the G2 and G1 genotypes already existed prior to the H1N1 outbreak of 2009, but they continued to circulate and evolve in America and other countries or regions during 2009 and 2010. Although the relative role of the NS1 gene mutation in the spread of 2009 H1N1 remains a matter for debate, this study contributes to a better understanding of the evolution and worldwide geographical spread of 2009 H1N1. Although the mutation at the residue 123 of the NS1 gene has been reported previously [25], its functions in virus replication and transmission remain unknown. Future studies are necessary to elucidate the effects of the V123I mutation on the binding capabilities of the NS and M proteins and on transmission between humans.

## Conclusions

All gene segments of the 2009 H1N1 virus, except for NS2, were under negative selection. Sequence analysis indicated that the G2 genotype, isolated mainly from Mexico, was likely the original type in this pandemic. The geographic distributions of the two viral genotypes had distinctive clustering features. These results will provide additional insight into the genetics and evolution of human pandemic influenza H1N1.

## Supporting Information

Figure S1 **Distribution of G1, and G2 type viruses in USA (A), Europe(B) and China (C).**
(DOCX)

Table S1 **Representative G2 genotypes of the pandemic H1N1/2009 human Influenza A Viruses from different countries or regions.**
(DOC)

Table S2 **G1 genotypes of the pandemic H1N1/2009 human Influenza A Viruses from different countries or regions.**
(DOCX)

Table S3 **The number of NS1,NS2,PA,NP,PB1,PB2,NA,HA,M1, and M2 gene sequence of novel swine-origin influenza virus A (H1N1) from April 2009 to May 2010 were used to calculate selection pressures.**
(DOCX)
