# Supplementary figures and images for: Evolutionary Characterization of the Pandemic H1N1/2009 Influenza Virus in Humans Based on Non-Structural Genes
Source: PLoS One. 2013 Feb 13;8(2):e56201. doi: 10.1371/journal.pone.0056201 (PMC3572024; doi:10.1371/journal.pone.0056201)

**Figure S1.** Distribution of G1, and G2 type viruses in USA (A), Europe(B) and China (C).


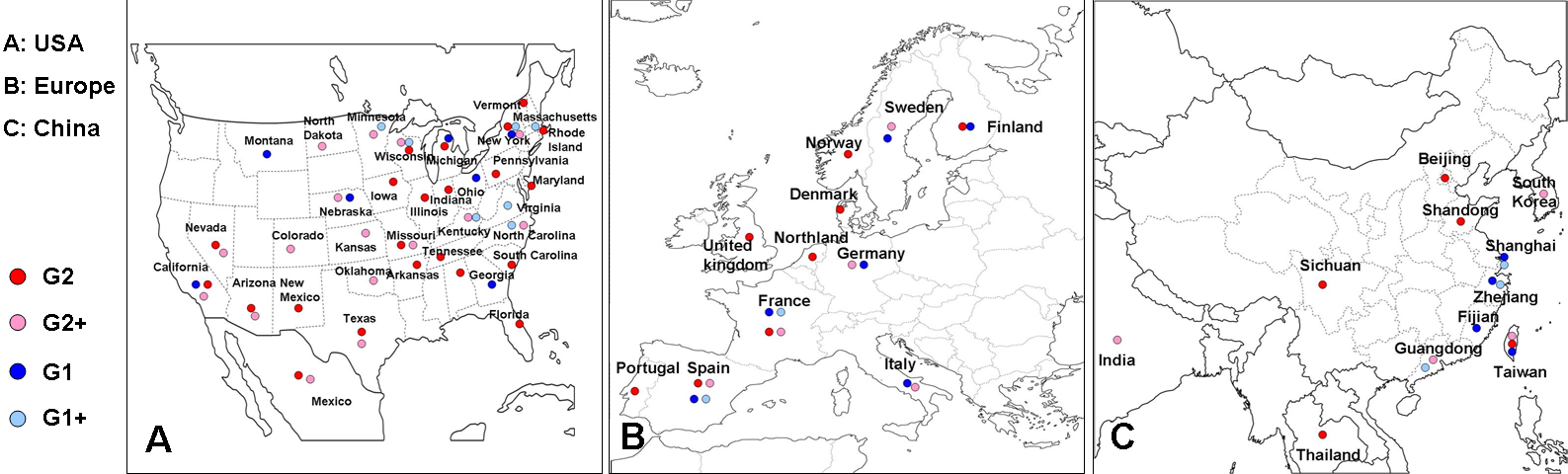

Supplement: Figure S1 — Distribution of G1, and G2 type viruses in USA (A), Europe(B) and China (C). (DOCX) [file pone.0056201.s001.docx]
