# Supplementary material for: Evolutionary Characterization of the Pandemic H1N1/2009 Influenza Virus in Humans Based on Non-Structural Genes
Source: PLoS One. 2013 Feb 13;8(2):e56201. doi: 10.1371/journal.pone.0056201 (PMC3572024; doi:10.1371/journal.pone.0056201)
Supplement: Table S1 — Representative G2 genotypes of the pandemic H1N1/2009 human Influenza A Viruses from different countries or regions. (DOC) [file pone.0056201.s002.doc]

**Table S1.** Representative G2 genotypes of the pandemic H1N1/2009 human Influenza A Viruses from different countries or regions.

| **Accession** | [**Segment**](../Sort%20by%20genome%20segment) | [**Country**](../Sort%20by%20collection%20country) | [**Date**](../Sort%20by%20collection%20date) | [**Virus name**](../Sort%20by%20virus%20name) |  |
| --- | --- | --- | --- | --- | --- |
| [CY049911](http://www.ncbi.nlm.nih.gov/entrez/viewer.fcgi?val=CY049911) | 8 (NS) | Dominican Republic | 2009-5-23 | A/Santo Domingo/0574/2009 | G2 |
| [GQ166657](http://www.ncbi.nlm.nih.gov/entrez/viewer.fcgi?val=GQ166657) | 8 (NS) | United Kingdom | 2009-4-28 | A/England/195/2009 | G2 |
| [GQ169304](http://www.ncbi.nlm.nih.gov/entrez/viewer.fcgi?val=GQ169304) | 8 (NS) | Japan | 2009-5-8 | A/Narita/1/2009 | G2 |
| [GQ166749](http://www.ncbi.nlm.nih.gov/entrez/viewer.fcgi?val=GQ166749) | 8 (NS) | Portugal | Apr-09 | A/Lisboa/26/2009 | G2 |
| [CY046945](http://www.ncbi.nlm.nih.gov/entrez/viewer.fcgi?val=CY046945) | 8 (NS) | Netherlands | 2009-4-29 | A/Netherlands/602/2009 | G2 |
| [FJ982433](http://www.ncbi.nlm.nih.gov/entrez/viewer.fcgi?val=FJ982433) | 8 (NS) | Denmark | 2009-4-29 | A/Denmark/513/2009 | G2 |
| [GQ166756](http://www.ncbi.nlm.nih.gov/entrez/viewer.fcgi?val=GQ166756) | 8 (NS) | Israel | Apr-09 | A/Israel/644/2009 | G2 |
| [CY040876](http://www.ncbi.nlm.nih.gov/entrez/viewer.fcgi?val=CY040876) | 8 (NS) | Norway | 2009-5-7 | A/Norway/1168/2009 | G2 |
| [GQ229379](http://www.ncbi.nlm.nih.gov/entrez/viewer.fcgi?val=GQ229379) | 8 (NS) | Thailand | 2009-5-6 | A/Thailand/104/2009 | G2 |
| GQ166230 | 8 (NS) | China | 2009-5-10 | A/Sichuan/1/2009 | G2 |
| CY044224 | 8 (NS) | Taiwan | 2009-5-28 | A/Taiwan/T1773/2009 | G2 |
| GQ283489 | 8 (NS) | Finland | 2009-5-26 | A/Finland/554/2009 | G2 |
| GQ214148 | 8 (NS) | France | 2009-4-30 | A/Paris/2580/2009 | G2 |
| [CY065774](http://www.ncbi.nlm.nih.gov/entrez/viewer.fcgi?val=CY065774) | 8 (NS) | Canada | Apr-09 | A/Canada/GFA0402/2009 | G2 |
| [FJ985757](http://www.ncbi.nlm.nih.gov/entrez/viewer.fcgi?val=FJ985757) | 8 (NS) | Spain | 2009-4-26 | A/Valencia/GP4/2009 | G2 |
| [GQ149658](http://www.ncbi.nlm.nih.gov/entrez/viewer.fcgi?val=GQ149658) | 8 (NS) | Mexico | 2009-4-3 | A/Mexico/4108/2009 | G2 |
| [FJ969514](http://www.ncbi.nlm.nih.gov/entrez/viewer.fcgi?val=FJ969514) | 8 (NS) | USA | 2009-4-1 | A/California/04/2009 | G2 |
| [GQ117106](http://www.ncbi.nlm.nih.gov/entrez/viewer.fcgi?val=GQ117106) | 8 (NS) | USA | Apr-09 | A/Nebraska/02/2009 | G2 |
| [GQ377090](http://www.ncbi.nlm.nih.gov/entrez/viewer.fcgi?val=GQ377090) | 8 (NS) | USA | 2009-4-27 | A/Colorado/03/2009 | G2 |
| [CY073354](http://www.ncbi.nlm.nih.gov/entrez/viewer.fcgi?val=CY073354) | 8 (NS) | USA | 2009-4-30 | A/San Diego/WR1629P/2009 | G2 |
| [CY073362](http://www.ncbi.nlm.nih.gov/entrez/viewer.fcgi?val=CY073362) | 8 (NS) | USA | 2009-5-8 | A/San Diego/WR1630P/2009 | G2 |
| [GQ160551](http://www.ncbi.nlm.nih.gov/entrez/viewer.fcgi?val=GQ160551) | 8 (NS) | USA | Apr-09 | A/New Jersey/04/2009 | G2 |
| [GQ160559](http://www.ncbi.nlm.nih.gov/entrez/viewer.fcgi?val=GQ160559) | 8 (NS) | USA | Apr-09 | A/New Jersey/02/2009 | G2 |
| [GQ200219](http://www.ncbi.nlm.nih.gov/entrez/viewer.fcgi?val=GQ200219) | 8 (NS) | USA | Apr-09 | A/Minnesota/03/2009 | G2 |
| [GQ117072](http://www.ncbi.nlm.nih.gov/entrez/viewer.fcgi?val=GQ117072) | 8 (NS) | USA | 2009-4-27 | A/Minnesota/02/2009 | G2 |
| [FJ981620](http://www.ncbi.nlm.nih.gov/entrez/viewer.fcgi?val=FJ981620) | 8 (NS) | USA | 2009-4-14 | A/Texas/04/2009 | G2 |
| [CY044247](http://www.ncbi.nlm.nih.gov/entrez/viewer.fcgi?val=CY044247) | 8 (NS) | USA | 2009-4-15 | A/San Antonio/PR922/2009 | G2 |
| [FJ966966](http://www.ncbi.nlm.nih.gov/entrez/viewer.fcgi?val=FJ966966) | 8 (NS) | USA | 2009-4-15 | A/Texas/05/2009 | G2 |
| [FJ981611](http://www.ncbi.nlm.nih.gov/entrez/viewer.fcgi?val=FJ981611) | 8 (NS) | USA | 2009-4-15 | A/Texas/05/2009 | G2 |
| [FJ984382](http://www.ncbi.nlm.nih.gov/entrez/viewer.fcgi?val=FJ984382) | 8 (NS) | USA | 2009-4-23 | A/Texas/06/2009 | G2 |
| [GQ168853](http://www.ncbi.nlm.nih.gov/entrez/viewer.fcgi?val=GQ168853) | 8 (NS) | USA | 2009-4-24 | A/Texas/08/2009 | G2 |
| [GQ221793](http://www.ncbi.nlm.nih.gov/entrez/viewer.fcgi?val=GQ221793) | 8 (NS) | USA | 2009-4-28 | A/Oklahoma/01/2009 | G2 |
| [CY044239](http://www.ncbi.nlm.nih.gov/entrez/viewer.fcgi?val=CY044239) | 8 (NS) | USA | 2009-4-14 | A/San Antonio/PR921/2009 | G2 |
| [CY044255](http://www.ncbi.nlm.nih.gov/entrez/viewer.fcgi?val=CY044255) | 8 (NS) | USA | 2009-4-23 | A/San Antonio/PR923/2009 | G2 |
| [GQ122093](http://www.ncbi.nlm.nih.gov/entrez/viewer.fcgi?val=GQ122093) | 8 (NS) | USA | 2009-4-15 | A/Texas/15/2009 | G2 |
| [GQ339883](http://www.ncbi.nlm.nih.gov/entrez/viewer.fcgi?val=GQ339883) | 8 (NS) | Mexico | 2009-4-15 | A/Mexico/4283/2009 | G2 |
| [GQ303343](http://www.ncbi.nlm.nih.gov/entrez/viewer.fcgi?val=GQ303343) | 8 (NS) | Mexico | 2009-4-20 | A/Mexico/4595/2009 | G2 |
| [GQ465725](http://www.ncbi.nlm.nih.gov/entrez/viewer.fcgi?val=GQ465725) | 8 (NS) | Canada | 2009-4-26 | A/Canada-PQ/RV1586/2009 | G2 |
| [FJ971074](http://www.ncbi.nlm.nih.gov/entrez/viewer.fcgi?val=FJ971074) | 8 (NS) | USA | 2009-4-16 | A/California/06/2009 | G2 |
| [CY043098](http://www.ncbi.nlm.nih.gov/entrez/viewer.fcgi?val=CY043098) | 8 (NS) | USA | 2009-4-21 | A/Brawley/40082/2009 | G2 |
| [CY050071](http://www.ncbi.nlm.nih.gov/entrez/viewer.fcgi?val=CY050071) | 8 (NS) | USA | 2009-4-28 | A/Cherry Point/WR1355/2009 | G2 |
| [CY073398](http://www.ncbi.nlm.nih.gov/entrez/viewer.fcgi?val=CY073398) | 8 (NS) | USA | 2009-4-28 | A/Brawley/WR1635P/2009 | G2 |
| [GQ323522](http://www.ncbi.nlm.nih.gov/entrez/viewer.fcgi?val=GQ323522) | 8 (NS) | USA | 2009-4-29 | A/Nevada/05/2009 | G2 |
| [CY073406](http://www.ncbi.nlm.nih.gov/entrez/viewer.fcgi?val=CY073406) | 8 (NS) | USA | 2009-4-29 | A/Brawley/WR1636P/2009 | G2 |
| [CY049832](http://www.ncbi.nlm.nih.gov/entrez/viewer.fcgi?val=CY049832) | 8 (NS) | USA | 2009-4-30 | A/Cherry Point/WR0080/2009 | G2 |
| [GQ465727](http://www.ncbi.nlm.nih.gov/entrez/viewer.fcgi?val=GQ465727) | 8 (NS) | Canada | 2009-5-1 | A/Canada-AB/RV1644/2009 | G2 |
| [CY073382](http://www.ncbi.nlm.nih.gov/entrez/viewer.fcgi?val=CY073382) | 8 (NS) | USA | 2009-5-1 | A/San Diego/WR1633P/2009 | G2 |
| [CY073430](http://www.ncbi.nlm.nih.gov/entrez/viewer.fcgi?val=CY073430) | 8 (NS) | USA | 2009-5-2 | A/San Diego/WR1641P/2009 | G2 |
| [CY054299](http://www.ncbi.nlm.nih.gov/entrez/viewer.fcgi?val=CY054299) | 8 (NS) | Mexico | 2009-5-2 | A/Mexico City/MCIG01/2009 | G2 |
| [CY049871](http://www.ncbi.nlm.nih.gov/entrez/viewer.fcgi?val=CY049871) | 8 (NS) | USA | 2009-5-2 | A/Cherry Point/WR0101/2009 | G2 |
| [CY049879](http://www.ncbi.nlm.nih.gov/entrez/viewer.fcgi?val=CY049879) | 8 (NS) | USA | 2009-5-2 | A/Cherry Point/WR0102/2009 | G2 |
| [CY045486](http://www.ncbi.nlm.nih.gov/entrez/viewer.fcgi?val=CY045486) | 8 (NS) | Germany | 2009-5-4 | A/Sachsen-Anhalt/101/2009 | G2 |
| [CY049847](http://www.ncbi.nlm.nih.gov/entrez/viewer.fcgi?val=CY049847) | 8 (NS) | USA | 2009-5-4 | A/Craven/WR0093/2009 | G2 |
| [CY049824](http://www.ncbi.nlm.nih.gov/entrez/viewer.fcgi?val=CY049824) | 8 (NS) | USA | 2009-5-5 | A/Craven/WR0019/2009 | G2 |
| [CY045494](http://www.ncbi.nlm.nih.gov/entrez/viewer.fcgi?val=CY045494) | 8 (NS) | Germany | 2009-5-6 | A/Sachsen-Anhalt/97/2009 | G2 |
| [GQ329103](http://www.ncbi.nlm.nih.gov/entrez/viewer.fcgi?val=GQ329103) | 8 (NS) | France | 2009-5-17 | A/Paris/2650/2009 | G2 |
| [GU136020](http://www.ncbi.nlm.nih.gov/entrez/viewer.fcgi?val=GU136020) | 8 (NS) | Japan | 2009-5-26 | A/Shizuoka-C/97/2009 | G2 |
| [CY043090](http://www.ncbi.nlm.nih.gov/entrez/viewer.fcgi?val=CY043090) | 8 (NS) | USA | 2009-4-22 | A/Brawley/40081/2009 | G2 |
| [GQ168856](http://www.ncbi.nlm.nih.gov/entrez/viewer.fcgi?val=GQ168856) | 8 (NS) | USA | 2009-4-24 | A/Kansas/02/2009 | G2 |
| [GQ117030](http://www.ncbi.nlm.nih.gov/entrez/viewer.fcgi?val=GQ117030) | 8 (NS) | USA | 2009-4-25 | A/Texas/09/2009 | G2 |
| [GQ160572](http://www.ncbi.nlm.nih.gov/entrez/viewer.fcgi?val=GQ160572) | 8 (NS) | USA | 2009-4-26 | A/Texas/22/2009 | G2 |
| [GQ117061](http://www.ncbi.nlm.nih.gov/entrez/viewer.fcgi?val=GQ117061) | 8 (NS) | USA | 2009-4-24 | A/Kansas/03/2009 | G2 |
| [GQ396593](http://www.ncbi.nlm.nih.gov/entrez/viewer.fcgi?val=GQ396593) | 8 (NS) | Spain | 2009-4-29 | A/Andalucia/GP251/2009 | G2 |
| [CY050178](http://www.ncbi.nlm.nih.gov/entrez/viewer.fcgi?val=CY050178) | 8 (NS) | Mexico | 2009-5-9 | A/Mexico City/015/2009 | G2 |
| [GQ303354](http://www.ncbi.nlm.nih.gov/entrez/viewer.fcgi?val=GQ303354) | 8 (NS) | Mexico | 2009-4-26 | A/Mexico/6882/2009 | G2 |
| [GQ323440](http://www.ncbi.nlm.nih.gov/entrez/viewer.fcgi?val=GQ323440) | 8 (NS) | USA | 2009-4-26 | A/Texas/19/2009 | G2 |
| [GQ396588](http://www.ncbi.nlm.nih.gov/entrez/viewer.fcgi?val=GQ396588) | 8 (NS) | Spain | 2009-4-26 | A/Madrid/GP62/2009 | G2 |
| [GQ396561](http://www.ncbi.nlm.nih.gov/entrez/viewer.fcgi?val=GQ396561) | 8 (NS) | Spain | 2009-4-29 | A/Andalucia/GP286/2009 | G2 |
| [GQ396549](http://www.ncbi.nlm.nih.gov/entrez/viewer.fcgi?val=GQ396549) | 8 (NS) | Spain | 2009-5-1 | A/Andalucia/GP327/2009 | G2 |
| [GQ396533](http://www.ncbi.nlm.nih.gov/entrez/viewer.fcgi?val=GQ396533) | 8 (NS) | Spain | 2009-5-1 | A/CastillaLaMancha/GP369/2009 | G2 |
| [GQ402271](http://www.ncbi.nlm.nih.gov/entrez/viewer.fcgi?val=GQ402271) | 8 (NS) | Mexico | 2009-4-27 | A/Mexico/InDRE13547/2009 | G2 |
| [CY041110](http://www.ncbi.nlm.nih.gov/entrez/viewer.fcgi?val=CY041110) | 8 (NS) | USA | 2009-4-28 | A/New York/3178/2009 | G2 |
| [GQ160577](http://www.ncbi.nlm.nih.gov/entrez/viewer.fcgi?val=GQ160577) | 8 (NS) | USA | 2009-4-28 | A/Washington/09/2009 | G2 |
| [GQ323504](http://www.ncbi.nlm.nih.gov/entrez/viewer.fcgi?val=GQ323504) | 8 (NS) | USA | 2009-5-7 | A/Washington/14/2009 | G2 |
| [GQ221698](http://www.ncbi.nlm.nih.gov/entrez/viewer.fcgi?val=GQ221698) | 8 (NS) | China | 2009-5-17 | A/GuangzhouSB/01/2009 | G2 |
| [GQ223447](http://www.ncbi.nlm.nih.gov/entrez/viewer.fcgi?val=GQ223447) | 8 (NS) | China | 2009-5-18 | A/GuangzhouSB/01/2009 | G2 |
| [HQ011407](http://www.ncbi.nlm.nih.gov/entrez/viewer.fcgi?val=HQ011407) | 8 (NS) | China | 2009-5-18 | A/Guangdong/01/2009 | G2 |
| [CY064795](http://www.ncbi.nlm.nih.gov/entrez/viewer.fcgi?val=CY064795) | 8 (NS) | China | 2009-5-28 | A/Guangdong/SB1/2009 | G2 |
| [HM780474](http://www.ncbi.nlm.nih.gov/entrez/viewer.fcgi?val=HM780474) | 8 (NS) | China | 2009-5-29 | A/Guangdong/06/2009 | G2 |
| [GQ402272](http://www.ncbi.nlm.nih.gov/entrez/viewer.fcgi?val=GQ402272) | 8 (NS) | Mexico | 2009-4-28 | A/Mexico/InDRE13551/2009 | G2 |
| [GQ214154](http://www.ncbi.nlm.nih.gov/entrez/viewer.fcgi?val=GQ214154) | 8 (NS) | France | 2009-4-29 | A/Paris/2573/2009 | G2 |
| [CY047314](http://www.ncbi.nlm.nih.gov/entrez/viewer.fcgi?val=CY047314) | 8 (NS) | USA | 2009-4-29 | A/New York/3074/2009 | G2 |
| [CY046311](http://www.ncbi.nlm.nih.gov/entrez/viewer.fcgi?val=CY046311) | 8 (NS) | USA | 2009-4-29 | A/Wisconsin/629-D01642/2009 | G2 |
| [CY050332](http://www.ncbi.nlm.nih.gov/entrez/viewer.fcgi?val=CY050332) | 8 (NS) | USA | 2009-5-12 | A/Wisconsin/629-D01851/2009 | G2 |
| [CY041625](http://www.ncbi.nlm.nih.gov/entrez/viewer.fcgi?val=CY041625) | 8 (NS) | USA | 2009-4-30 | A/New York/3100/2009 | G2 |
| [CY046359](http://www.ncbi.nlm.nih.gov/entrez/viewer.fcgi?val=CY046359) | 8 (NS) | USA | 2009-4-30 | A/Wisconsin/629-D01529/2009 | G2 |
| [CY046295](http://www.ncbi.nlm.nih.gov/entrez/viewer.fcgi?val=CY046295) | 8 (NS) | USA | 2009-5-1 | A/Wisconsin/629-D01817/2009 | G2 |
| [CY046239](http://www.ncbi.nlm.nih.gov/entrez/viewer.fcgi?val=CY046239) | 8 (NS) | USA | 2009-5-2 | A/Wisconsin/629-D01735/2009 | G2 |
| [CY046887](http://www.ncbi.nlm.nih.gov/entrez/viewer.fcgi?val=CY046887) | 8 (NS) | USA | 2009-5-3 | A/Wisconsin/629-D00498/2009 | G2 |
| [CY046215](http://www.ncbi.nlm.nih.gov/entrez/viewer.fcgi?val=CY046215) | 8 (NS) | USA | 2009-5-3 | A/Wisconsin/629-D00869/2009 | G2 |
| [CY046503](http://www.ncbi.nlm.nih.gov/entrez/viewer.fcgi?val=CY046503) | 8 (NS) | USA | 2009-5-4 | A/Wisconsin/629-D00378/2009 | G2 |
| [CY050146](http://www.ncbi.nlm.nih.gov/entrez/viewer.fcgi?val=CY050146) | 8 (NS) | USA | 2009-5-4 | A/Wisconsin/629-D01058/2009 | G2 |
| [CY050448](http://www.ncbi.nlm.nih.gov/entrez/viewer.fcgi?val=CY050448) | 8 (NS) | USA | 2009-5-10 | A/Wisconsin/629-D01482/2009 | G2 |
| [CY046599](http://www.ncbi.nlm.nih.gov/entrez/viewer.fcgi?val=CY046599) | 8 (NS) | USA | 2009-5-10 | A/Wisconsin/629-D01482/2009 | G2 |
| [CY046687](http://www.ncbi.nlm.nih.gov/entrez/viewer.fcgi?val=CY046687) | 8 (NS) | USA | 2009-5-14 | A/Wisconsin/629-D00978/2009 | G2 |
| [CY046631](http://www.ncbi.nlm.nih.gov/entrez/viewer.fcgi?val=CY046631) | 8 (NS) | USA | 2009-5-15 | A/Wisconsin/629-D01839/2009 | G2 |
| [CY050384](http://www.ncbi.nlm.nih.gov/entrez/viewer.fcgi?val=CY050384) | 8 (NS) | USA | 2009-5-15 | A/Wisconsin/629-D01295/2009 | G2 |
| [CY050392](http://www.ncbi.nlm.nih.gov/entrez/viewer.fcgi?val=CY050392) | 8 (NS) | USA | 2009-5-15 | A/Wisconsin/629-D01839/2009 | G2 |
| [CY046711](http://www.ncbi.nlm.nih.gov/entrez/viewer.fcgi?val=CY046711) | 8 (NS) | USA | 2009-5-20 | A/Wisconsin/629-D01787/2009 | G2 |
| [CY073370](http://www.ncbi.nlm.nih.gov/entrez/viewer.fcgi?val=CY073370) | 8 (NS) | USA | 2009-4-30 | A/San Diego/WR1631P/2009 | G2 |
| [GQ214142](http://www.ncbi.nlm.nih.gov/entrez/viewer.fcgi?val=GQ214142) | 8 (NS) | France | 2009-4-30 | A/Paris/2590/2009 | G2 |
| [GQ232008](http://www.ncbi.nlm.nih.gov/entrez/viewer.fcgi?val=GQ232008) | 8 (NS) | USA | 2009-4-30 | A/Kentucky/05/2009 | G2 |
| [CY064704](http://www.ncbi.nlm.nih.gov/entrez/viewer.fcgi?val=CY064704) | 8 (NS) | Mexico | 2009-4-30 | A/Mexico City/019/2009 | G2 |
| [CY064744](http://www.ncbi.nlm.nih.gov/entrez/viewer.fcgi?val=CY064744) | 8 (NS) | Mexico | 2009-4-30 | A/Mexico City/024/2009 | G2 |
| [GU292395](http://www.ncbi.nlm.nih.gov/entrez/viewer.fcgi?val=GU292395) | 8 (NS) | India | May-09 | A/Hyd/NIV51/2009 | G2 |
| [CY041641](http://www.ncbi.nlm.nih.gov/entrez/viewer.fcgi?val=CY041641) | 8 (NS) | USA | 2009-5-1 | A/New York/3307/2009 | G2 |
| [CY046975](http://www.ncbi.nlm.nih.gov/entrez/viewer.fcgi?val=CY046975) | 8 (NS) | USA | 2009-5-1 | A/New York/3443/2009 | G2 |
| [CY046319](http://www.ncbi.nlm.nih.gov/entrez/viewer.fcgi?val=CY046319) | 8 (NS) | USA | 2009-5-1 | A/Wisconsin/629-D01308/2009 | G2 |
| [CY046439](http://www.ncbi.nlm.nih.gov/entrez/viewer.fcgi?val=CY046439) | 8 (NS) | USA | 2009-5-2 | A/Wisconsin/629-D02262/2009 | G2 |
| [CY046399](http://www.ncbi.nlm.nih.gov/entrez/viewer.fcgi?val=CY046399) | 8 (NS) | USA | 2009-5-2 | A/Wisconsin/629-D01189/2009 | G2 |
| [CY064720](http://www.ncbi.nlm.nih.gov/entrez/viewer.fcgi?val=CY064720) | 8 (NS) | Mexico | 2009-5-1 | A/Mexico City/021/2009 | G2 |
| [GQ249335](http://www.ncbi.nlm.nih.gov/entrez/viewer.fcgi?val=GQ249335) | 8 (NS) | France | 2009-5-1 | A/Paris/2591/2009 | G2 |
| [CY073390](http://www.ncbi.nlm.nih.gov/entrez/viewer.fcgi?val=CY073390) | 8 (NS) | USA | 2009-5-2 | A/San Diego/WR1634P/2009 | G2 |
| [GQ131026](http://www.ncbi.nlm.nih.gov/entrez/viewer.fcgi?val=GQ131026) | 8 (NS) | South Korea | 2009-5-2 | A/Korea/01/2009 | G2 |
| [GQ223430](http://www.ncbi.nlm.nih.gov/entrez/viewer.fcgi?val=GQ223430) | 8 (NS) | Japan | 2009-5-16 | A/Osaka/1/2009 | G2 |
| [GQ223431](http://www.ncbi.nlm.nih.gov/entrez/viewer.fcgi?val=GQ223431) | 8 (NS) | Japan | 2009-5-16 | A/Osaka/2/2009 | G2 |
| [GQ223432](http://www.ncbi.nlm.nih.gov/entrez/viewer.fcgi?val=GQ223432) | 8 (NS) | Japan | 2009-5-16 | A/Osaka-C/1/2009 | G2 |
| [GQ223433](http://www.ncbi.nlm.nih.gov/entrez/viewer.fcgi?val=GQ223433) | 8 (NS) | Japan | 2009-5-17 | A/Osaka-C/2/2009 | G2 |
| [GQ223428](http://www.ncbi.nlm.nih.gov/entrez/viewer.fcgi?val=GQ223428) | 8 (NS) | Japan | 2009-5-17 | A/Hyogo/2/2009 | G2 |
| [GQ267835](http://www.ncbi.nlm.nih.gov/entrez/viewer.fcgi?val=GQ267835) | 8 (NS) | Japan | 2009-5-18 | A/Himeji/1/2009 | G2 |
| [GQ300863](http://www.ncbi.nlm.nih.gov/entrez/viewer.fcgi?val=GQ300863) | 8 (NS) | Japan | 2009-5-19 | A/Shiga/2/2009 | G2 |
| [GQ267842](http://www.ncbi.nlm.nih.gov/entrez/viewer.fcgi?val=GQ267842) | 8 (NS) | Japan | 2009-5-21 | A/Sakai/1/2009 | G2 |
| [GQ267842](http://www.ncbi.nlm.nih.gov/entrez/viewer.fcgi?val=GQ267842) | 8 (NS) | Japan | 2009-5-21 | A/Sakai/1/2009 | G2 |
| [CY064421](http://www.ncbi.nlm.nih.gov/entrez/viewer.fcgi?val=CY064421) | 8 (NS) | Mexico | 2009-5-2 | A/Mexico City/CIA11/2009 | G2 |
| [CY064414](http://www.ncbi.nlm.nih.gov/entrez/viewer.fcgi?val=CY064414) | 8 (NS) | Mexico | 2009-5-3 | A/Mexico City/CIA10/2009 | G2 |
| [CY050851](http://www.ncbi.nlm.nih.gov/entrez/viewer.fcgi?val=CY050851) | 8 (NS) | Mexico | 2009-5-9 | A/Mexico City/007/2009 | G2 |
| [CY062544](http://www.ncbi.nlm.nih.gov/entrez/viewer.fcgi?val=CY062544) | 8 (NS) | Mexico | 2009-5-16 | A/Mexico City/CIA9/2009 | G2 |
| [CY046343](http://www.ncbi.nlm.nih.gov/entrez/viewer.fcgi?val=CY046343) | 8 (NS) | USA | 2009-5-2 | A/Wisconsin/629-D02455/2009 | G2 |
| [CY046287](http://www.ncbi.nlm.nih.gov/entrez/viewer.fcgi?val=CY046287) | 8 (NS) | USA | 2009-5-3 | A/Wisconsin/629-D02473/2009 | G2 |
| [CY046375](http://www.ncbi.nlm.nih.gov/entrez/viewer.fcgi?val=CY046375) | 8 (NS) | USA | 2009-5-3 | A/Wisconsin/629-D01705/2009 | G2 |
| [GQ251039](http://www.ncbi.nlm.nih.gov/entrez/viewer.fcgi?val=GQ251039) | 8 (NS) | Italy | 2009-5-3 | A/Italy/05/2009 | G2 |
| [GQ402276](http://www.ncbi.nlm.nih.gov/entrez/viewer.fcgi?val=GQ402276) | 8 (NS) | Canada | 2009-5-4 | A/Canada-SK/RV1767/2009 | G2 |
| [CY046447](http://www.ncbi.nlm.nih.gov/entrez/viewer.fcgi?val=CY046447) | 8 (NS) | USA | 2009-5-4 | A/Wisconsin/629-D01226/2009 | G2 |
| [CY043175](http://www.ncbi.nlm.nih.gov/entrez/viewer.fcgi?val=CY043175) | 8 (NS) | USA | 2009-5-4 | A/New York/3460/2009 | G2 |
| [CY053146](http://www.ncbi.nlm.nih.gov/entrez/viewer.fcgi?val=CY053146) | 8 (NS) | USA | 2009-5-4 | A/Houston/22OS/2009 | G2 |
| [CY053154](http://www.ncbi.nlm.nih.gov/entrez/viewer.fcgi?val=CY053154) | 8 (NS) | USA | 2009-5-4 | A/Houston/22H/2009 | G2 |
| [CY046607](http://www.ncbi.nlm.nih.gov/entrez/viewer.fcgi?val=CY046607) | 8 (NS) | USA | 2009-5-7 | A/Wisconsin/629-D00487/2009 | G2 |
| [GQ232030](http://www.ncbi.nlm.nih.gov/entrez/viewer.fcgi?val=GQ232030) | 8 (NS) | USA | 2009-5-7 | A/Arizona/07/2009 | G2 |
| [GQ323539](http://www.ncbi.nlm.nih.gov/entrez/viewer.fcgi?val=GQ323539) | 8 (NS) | USA | 2009-5-8 | A/Texas/35/2009 | G2 |
| [CY050218](http://www.ncbi.nlm.nih.gov/entrez/viewer.fcgi?val=CY050218) | 8 (NS) | Mexico | 2009-5-8 | A/Mexico City/003/2009 | G2 |
| [CY043127](http://www.ncbi.nlm.nih.gov/entrez/viewer.fcgi?val=CY043127) | 8 (NS) | USA | 2009-5-8 | A/New York/3389/2009 | G2 |
| [CY046615](http://www.ncbi.nlm.nih.gov/entrez/viewer.fcgi?val=CY046615) | 8 (NS) | USA | 2009-5-9 | A/Wisconsin/629-D01055/2009 | G2 |
| [CY050242](http://www.ncbi.nlm.nih.gov/entrez/viewer.fcgi?val=CY050242) | 8 (NS) | Mexico | 2009-5-9 | A/Mexico City/010/2009 | G2 |
| [CY050258](http://www.ncbi.nlm.nih.gov/entrez/viewer.fcgi?val=CY050258) | 8 (NS) | Mexico | 2009-5-9 | A/Mexico City/012/2009 | G2 |
| [GQ323534](http://www.ncbi.nlm.nih.gov/entrez/viewer.fcgi?val=GQ323534) | 8 (NS) | USA | 2009-5-10 | A/Missouri/04/2009 | G2 |
| [GQ232059](http://www.ncbi.nlm.nih.gov/entrez/viewer.fcgi?val=GQ232059) | 8 (NS) | USA | 2009-5-11 | A/North Dakota/04/2009 | G2 |
| [CY046695](http://www.ncbi.nlm.nih.gov/entrez/viewer.fcgi?val=CY046695) | 8 (NS) | USA | 2009-5-16 | A/Wisconsin/629-D00434/2009 | G2 |
| [CY046767](http://www.ncbi.nlm.nih.gov/entrez/viewer.fcgi?val=CY046767) | 8 (NS) | USA | 2009-5-22 | A/Wisconsin/629-D02298/2009 | G2 |
| [CY052963](http://www.ncbi.nlm.nih.gov/entrez/viewer.fcgi?val=CY052963) | 8 (NS) | USA | 2009-5-13 | A/Houston/1H/2009 | G2 |
| [GQ223427](http://www.ncbi.nlm.nih.gov/entrez/viewer.fcgi?val=GQ223427) | 8 (NS) | Japan | 2009-5-17 | A/Hyogo/1/2009 | G2 |
| [GQ402280](http://www.ncbi.nlm.nih.gov/entrez/viewer.fcgi?val=GQ402280) | 8 (NS) | Canada | 2009-5-20 | A/Canada-MB/RV1964/2009 | G2 |
| [CY053194](http://www.ncbi.nlm.nih.gov/entrez/viewer.fcgi?val=CY053194) | 8 (NS) | USA | 2009-5-21 | A/Brownsville/29OS/2009 | G2 |
| [CY053202](http://www.ncbi.nlm.nih.gov/entrez/viewer.fcgi?val=CY053202) | 8 (NS) | USA | 2009-5-21 | A/Brownsville/30OS/2009 | G2 |
| [CY053051](http://www.ncbi.nlm.nih.gov/entrez/viewer.fcgi?val=CY053051) | 8 (NS) | USA | 2009-5-21 | A/Houston/9H/2009 | G2 |
| [CY044881](http://www.ncbi.nlm.nih.gov/entrez/viewer.fcgi?val=CY044881) | 8 (NS) | USA | 2009-5-21 | A/New York/3573/2009 | G2 |
| [CY046967](http://www.ncbi.nlm.nih.gov/entrez/viewer.fcgi?val=CY046967) | 8 (NS) | USA | 2009-5-21 | A/New York/3576/2009 | G2 |
| [CY044969](http://www.ncbi.nlm.nih.gov/entrez/viewer.fcgi?val=CY044969) | 8 (NS) | USA | 2009-5-21 | A/New York/3653/2009 | G2 |
| [GQ267854](http://www.ncbi.nlm.nih.gov/entrez/viewer.fcgi?val=GQ267854) | 8 (NS) | Japan | 2009-5-22 | A/Shiga/1/2009 | G2 |
| [CY044208](http://www.ncbi.nlm.nih.gov/entrez/viewer.fcgi?val=CY044208) | 8 (NS) | Taiwan | 2009-5-22 | A/Taiwan/T1338/2009 | G2 |
| [CY053210](http://www.ncbi.nlm.nih.gov/entrez/viewer.fcgi?val=CY053210) | 8 (NS) | USA | 2009-5-22 | A/Brownsville/31OS/2009 | G2 |
| [CY053218](http://www.ncbi.nlm.nih.gov/entrez/viewer.fcgi?val=CY053218) | 8 (NS) | USA | 2009-5-22 | A/Brownsville/31H/2009 | G2 |
| [GQ402281](http://www.ncbi.nlm.nih.gov/entrez/viewer.fcgi?val=GQ402281) | 8 (NS) | Canada | 2009-5-24 | A/Canada-MB/RV1975/2009 | G2 |
| [GQ402285](http://www.ncbi.nlm.nih.gov/entrez/viewer.fcgi?val=GQ402285) | 8 (NS) | Canada | 2009-5-29 | A/Canada-MB/RV2018/2009 | G2 |
| [CY045966](http://www.ncbi.nlm.nih.gov/entrez/viewer.fcgi?val=CY045966) | 8 (NS) | Canada | 2009-5-30 | A/Toronto/T5294/2009 | G2 |
| [CY051251](http://www.ncbi.nlm.nih.gov/entrez/viewer.fcgi?val=CY051251) | 8 (NS) | USA | 2009-5-26 | A/Wisconsin/629-S0035/2009 | G2 |
| [CY046959](http://www.ncbi.nlm.nih.gov/entrez/viewer.fcgi?val=CY046959) | 8 (NS) | USA | 2009-5-26 | A/New York/3654/2009 | G2 |
| [CY053258](http://www.ncbi.nlm.nih.gov/entrez/viewer.fcgi?val=CY053258) | 8 (NS) | USA | 2009-5-27 | A/Brownsville/36H/2009 | G2 |
| [CY046799](http://www.ncbi.nlm.nih.gov/entrez/viewer.fcgi?val=CY046799) | 8 (NS) | USA | 2009-5-28 | A/Wisconsin/629-D01026/2009 | G2 |
| [CY045009](http://www.ncbi.nlm.nih.gov/entrez/viewer.fcgi?val=CY045009) | 8 (NS) | USA | 2009-5-28 | A/New York/3709/2009 | G2 |
| [GQ360059](http://www.ncbi.nlm.nih.gov/entrez/viewer.fcgi?val=GQ360059) | 8 (NS) | Sweden | 2009-5-29 | A/Stockholm/34/2009 | G2 |
