# Supplementary material for: Evolutionary Characterization of the Pandemic H1N1/2009 Influenza Virus in Humans Based on Non-Structural Genes
Source: PLoS One. 2013 Feb 13;8(2):e56201. doi: 10.1371/journal.pone.0056201 (PMC3572024; doi:10.1371/journal.pone.0056201)
Supplement: Table S2 — G1 genotypes of the pandemic H1N1/2009 human Influenza A Viruses from different countries or regions. (DOCX) [file pone.0056201.s003.docx]

**Table S2.** G1 genotypes of the pandemic H1N1/2009 human Influenza A Viruses from different countries or regions.

| **Accession** | **Segment** | **Country** | **Date** | **Virus name** |  |
| --- | --- | --- | --- | --- | --- |
| [GQ232063](http://www.ncbi.nlm.nih.gov/entrez/viewer.fcgi?val=GQ232063) | 8 (NS) | USA | 2009-4-25 | A/New York/18/2009 | G1 |
| [FJ984349](http://www.ncbi.nlm.nih.gov/entrez/viewer.fcgi?val=FJ984349) | 8 (NS) | USA | 2009-4-25 | A/New York/18/2009 | G1 |
| [CY041118](http://www.ncbi.nlm.nih.gov/entrez/viewer.fcgi?val=CY041118) | 8 (NS) | USA | 2009-4-28 | A/New York/3193/2009 | G1 |
| [GQ168848](http://www.ncbi.nlm.nih.gov/entrez/viewer.fcgi?val=GQ168848) | 8 (NS) | USA | 2009-4-26 | A/Massachusetts/06/2009 | G1 |
| [GQ168874](http://www.ncbi.nlm.nih.gov/entrez/viewer.fcgi?val=GQ168874) | 8 (NS) | USA | 2009-4-26 | A/Massachusetts/07/2009 | G1 |
| [GQ396527](http://www.ncbi.nlm.nih.gov/entrez/viewer.fcgi?val=GQ396527) | 8 (NS) | Spain | 2009-5-13 | A/Madrid/GP523/2009 | G1 |
| [GQ160590](http://www.ncbi.nlm.nih.gov/entrez/viewer.fcgi?val=GQ160590) | 8 (NS) | USA | 2009-4-27 | A/Virginia/04/2009 | G1 |
| [CY041518](http://www.ncbi.nlm.nih.gov/entrez/viewer.fcgi?val=CY041518) | 8 (NS) | USA | 2009-4-27 | A/New York/3203/2009 | G1 |
| [CY040027](http://www.ncbi.nlm.nih.gov/entrez/viewer.fcgi?val=CY040027) | 8 (NS) | USA | 2009-4-27 | A/New York/3049/2009 | G1 |
| [CY040713](http://www.ncbi.nlm.nih.gov/entrez/viewer.fcgi?val=CY040713) | 8 (NS) | USA | 2009-4-28 | A/New York/3237/2009 | G1 |
| [CY041494](http://www.ncbi.nlm.nih.gov/entrez/viewer.fcgi?val=CY041494) | 8 (NS) | USA | 2009-4-28 | A/New York/3184/2009 | G1 |
| [CY040834](http://www.ncbi.nlm.nih.gov/entrez/viewer.fcgi?val=CY040834) | 8 (NS) | USA | 2009-4-29 | A/New York/3261/2009 | G1 |
| [CY049863](http://www.ncbi.nlm.nih.gov/entrez/viewer.fcgi?val=CY049863) | 8 (NS) | USA | 2009-5-2 | A/Cherry Point/WR0100/2009 | G1 |
| [GQ323466](http://www.ncbi.nlm.nih.gov/entrez/viewer.fcgi?val=GQ323466) | 8 (NS) | USA | 2009-5-4 | A/Kentucky/07/2009 | G1 |
| [CY043255](http://www.ncbi.nlm.nih.gov/entrez/viewer.fcgi?val=CY043255) | 8 (NS) | USA | 2009-5-16 | A/New York/3545/2009 | G1 |
| [CY071027](http://www.ncbi.nlm.nih.gov/entrez/viewer.fcgi?val=CY071027) | 8 (NS) | USA | 2009-5-31 | A/New York/NHRC0001/2009 | G1 |
| [CY043231](http://www.ncbi.nlm.nih.gov/entrez/viewer.fcgi?val=CY043231) | 8 (NS) | USA | 2009-5-17 | A/New York/3501/2009 | G1 |
| [CY044913](http://www.ncbi.nlm.nih.gov/entrez/viewer.fcgi?val=CY044913) | 8 (NS) | USA | 2009-5-18 | A/New York/3612/2009 | G1 |
| [CY047362](http://www.ncbi.nlm.nih.gov/entrez/viewer.fcgi?val=CY047362) | 8 (NS) | USA | 2009-5-19 | A/New York/3741/2009 | G1 |
| [CY043151](http://www.ncbi.nlm.nih.gov/entrez/viewer.fcgi?val=CY043151) | 8 (NS) | USA | 2009-5-20 | A/New York/3551/2009 | G1 |
| [GQ329085](http://www.ncbi.nlm.nih.gov/entrez/viewer.fcgi?val=GQ329085) | 8 (NS) | France | 2009-5-22 | A/Paris/2670/2009 | G1 |
| [CY045097](http://www.ncbi.nlm.nih.gov/entrez/viewer.fcgi?val=CY045097) | 8 (NS) | USA | 2009-5-22 | A/New York/3614/2009 | G1 |
| [GQ225353](http://www.ncbi.nlm.nih.gov/entrez/viewer.fcgi?val=GQ225353) | 8 (NS) | China | 2009-5-23 | A/Zhejiang/1/2009 | G1 |
| [GQ225361](http://www.ncbi.nlm.nih.gov/entrez/viewer.fcgi?val=GQ225361) | 8 (NS) | China | 2009-5-23 | A/Shanghai/1/2009 | G1 |
| [GQ253493](http://www.ncbi.nlm.nih.gov/entrez/viewer.fcgi?val=GQ253493) | 8 (NS) | China | 2009-5-24 | A/Shanghai/37T/2009 | G1 |
| [GQ290437](http://www.ncbi.nlm.nih.gov/entrez/viewer.fcgi?val=GQ290437) | 8 (NS) | China | 2009-5-28 | A/Shanghai/60T/2009 | G1 |
| [CY046703](http://www.ncbi.nlm.nih.gov/entrez/viewer.fcgi?val=CY046703) | 8 (NS) | USA | 2009-5-24 | A/Wisconsin/629-D01445/2009 | G1 |
| [CY044076](http://www.ncbi.nlm.nih.gov/entrez/viewer.fcgi?val=CY044076) | 8 (NS) | USA | 2009-5-26 | A/New York/3702/2009 | G1 |
| [CY045155](http://www.ncbi.nlm.nih.gov/entrez/viewer.fcgi?val=CY045155) | 8 (NS) | USA | 2009-5-26 | A/New York/3655/2009 | G1 |
| [CY049935](http://www.ncbi.nlm.nih.gov/entrez/viewer.fcgi?val=CY049935) | 8 (NS) | USA | 2009-5-27 | A/Craven/WR1001/2009 | G1 |
| [GQ232089](http://www.ncbi.nlm.nih.gov/entrez/viewer.fcgi?val=GQ232089) | 8 (NS) | China | 2009-5-27 | A/Guangdong/02/2009 | G1 |
| [HQ011422](http://www.ncbi.nlm.nih.gov/entrez/viewer.fcgi?val=HQ011422) | 8 (NS) | China | 2009-5-28 | A/Guangdong/45/2009 | G1 |
| [GQ244325](http://www.ncbi.nlm.nih.gov/entrez/viewer.fcgi?val=GQ244325) | 8 (NS) | China | 2009-5-29 | A/Guangdong/05/2009 | G1 |
| [GQ227549](http://www.ncbi.nlm.nih.gov/entrez/viewer.fcgi?val=GQ227549) | 8 (NS) | China | 2009-5-29 | A/Guangdong/03/2009 | G1 |
| [CY041975](http://www.ncbi.nlm.nih.gov/entrez/viewer.fcgi?val=CY041975) | 8 (NS) | Israel | Apr-09 | A/Israel/277/2009 | G1 |
| [CY048929](http://www.ncbi.nlm.nih.gov/entrez/viewer.fcgi?val=CY048929) | 8 (NS) | Malaysia | 2009-5-15 | A/Malaysia/820/2009 | G1 |
| [CY049072](http://www.ncbi.nlm.nih.gov/entrez/viewer.fcgi?val=CY049072) | 8 (NS) | Singapore | 2009-5-28 | A/Singapore/ON129/2009 | G1 |
| [GQ223439](http://www.ncbi.nlm.nih.gov/entrez/viewer.fcgi?val=GQ223439) | 8 (NS) | Finland | 2009-5-10 | A/Finland/553/2009 | G1 |
| [GQ288376](http://www.ncbi.nlm.nih.gov/entrez/viewer.fcgi?val=GQ288376) | 8 (NS) | China | 2009-5-28 | A/Fuzhou/01/2009 | G1 |
| [CY044200](http://www.ncbi.nlm.nih.gov/entrez/viewer.fcgi?val=CY044200) | 8 (NS) | Taiwan | 2009-5-19 | A/Taiwan/T0724/2009 | G1 |
| [GQ202724](http://www.ncbi.nlm.nih.gov/entrez/viewer.fcgi?val=GQ202724) | 8 (NS) | Russia | 2009-5-21 | A/Moscow/IIV01/2009 | G1 |
| [GQ283479](http://www.ncbi.nlm.nih.gov/entrez/viewer.fcgi?val=GQ283479) | 8 (NS) | Italy | 2009-5-27 | A/Italy/49/2009 | G1 |
| [GQ329109](http://www.ncbi.nlm.nih.gov/entrez/viewer.fcgi?val=GQ329109) | 8 (NS) | France | 2009-5-6 | A/Strasbourg/2609/2009 | G1 |
| [HM569663](http://www.ncbi.nlm.nih.gov/entrez/viewer.fcgi?val=HM569663) | 8 (NS) | Argentina | 2009-5-30 | A/Argentina/07-09GP/2009 | G1 |
| [GQ365678](http://www.ncbi.nlm.nih.gov/entrez/viewer.fcgi?val=GQ365678) | 8 (NS) | Germany | 2009-5-4 | A/Brandenburg/20/2009 | G1 |
| [CY065766](http://www.ncbi.nlm.nih.gov/entrez/viewer.fcgi?val=CY065766) | 8 (NS) | Canada | Apr-09 | A/British Columbia/GFA0401/2009 | G1 |
| [CY075087](http://www.ncbi.nlm.nih.gov/entrez/viewer.fcgi?val=CY075087) | 8 (NS) | Chile | 2009-5-23 | A/Chile/28/2009 | G1 |
| [GQ396565](http://www.ncbi.nlm.nih.gov/entrez/viewer.fcgi?val=GQ396565) | 8 (NS) | Spain | 2009-4-29 | A/Valencia/GP272/2009 | G1 |
| [GQ359770](http://www.ncbi.nlm.nih.gov/entrez/viewer.fcgi?val=GQ359770) | 8 (NS) | Sweden | 2009-5-26 | A/Stockholm/30/2009 | G1 |
| [FJ984356](http://www.ncbi.nlm.nih.gov/entrez/viewer.fcgi?val=FJ984356) | 8 (NS) | USA | 2009-4-24 | A/New York/31/2009 | G1 |
|  |  |  |  |  |  |
