# Supplementary material for: Evolutionary Characterization of the Pandemic H1N1/2009 Influenza Virus in Humans Based on Non-Structural Genes
Source: PLoS One. 2013 Feb 13;8(2):e56201. doi: 10.1371/journal.pone.0056201 (PMC3572024; doi:10.1371/journal.pone.0056201)
Supplement: Table S3 — The number of NS1,NS2,PA,NP,PB1,PB2,NA,HA,M1, and M2 gene sequence of novel swine-origin influenza virus A (H1N1) from April 2009 to May 2010 were used to calculate selection pressures. (DOCX) [file pone.0056201.s004.docx]

**Table S3.** The number of NS1,NS2,PA,NP,PB1,PB2,NA,HA,M1, and M2 gene sequence of novel swine-origin influenza virus A (H1N1) from April 2009 to May 2010 were used to calculate selection pressures.

|  | **2009** | | | | | | | | | **2010** |
| --- | --- | --- | --- | --- | --- | --- | --- | --- | --- | --- |
|  | **April** | **May** | **June** | **July** | **August** | **September** | **October** | **November** | **December** | **January to May** |
| **NS1** | 222 | 388 | 384 | 212 | 139 | 198 | 229 | 313 | 249 | 102 |
| **NS2** | 222 | 388 | 384 | 212 | 139 | 198 | 229 | 313 | 249 | 102 |
| **PA** | 267 | 383 | 402 | 234 | 146 | 187 | 211 | 307 | 253 | 102 |
| **NP** | 347 | 403 | 403 | 230 | 147 | 190 | 213 | 308 | 253 | 103 |
| **PB1** | 283 | 379 | 372 | 202 | 131 | 180 | 199 | 199 | 199 | 199 |
| **PB2** | 270 | 383 | 380 | 217 | 140 | 186 | 214 | 307 | 254 | 103 |
| **HA** | 433 | 473 | 486 | 435 | 279 | 299 | 309 | 478 | 300 | 188 |
| **M1** | 378 | 435 | 413 | 226 | 145 | 205 | 275 | 331 | 264 | 108 |
| **M2** | 366 | 429 | 401 | 210 | 141 | 198 | 273 | 321 | 264 | 108 |
| **NA** | 413 | 471 | 466 | 348 | 209 | 264 | 297 | 411 | 283 | 121 |
